# Integrated transcriptional‐phenotypic analysis captures systemic immunomodulation following antiangiogenic therapy in renal cell carcinoma patients

**DOI:** 10.1002/ctm2.434

**Published:** 2021-06-20

**Authors:** Darawan Rinchai, Elena Verzoni, Veronica Huber, Agata Cova, Paola Squarcina, Loris De Cecco, Filippo de Braud, Raffaele Ratta, Matteo Dugo, Luca Lalli, Viviana Vallacchi, Monica Rodolfo, Jessica Roelands, Chiara Castelli, Damien Chaussabel, Giuseppe Procopio, Davide Bedognetti, Licia Rivoltini

**Affiliations:** ^1^ Cancer Research Department Sidra Medicine Doha Qatar; ^2^ Medical Oncology Department Fondazione IRCCS Istituto Nazionale dei Tumori Milan Italy; ^3^ Unit of Immunotherapy of Human Tumors Fondazione IRCCS Istituto Nazionale dei Tumori Milan Italy; ^4^ Platform of Integrated Biology Fondazione IRCCS Istituto Nazionale dei Tumori Milan Italy; ^5^ Medical Oncology Department Hopital Foch Suresnes France; ^6^ Immunology Research Department Sidra Medicine Doha Qatar; ^7^ Dipartimento di Medicina Interna e Specialità Mediche Università degli Studi di Genova Genova Italy; ^8^ College of Health and Life Sciences Hamad Bin Khalifa University Doha Qatar

**Keywords:** antiangiogenics, bioinformatics, blood transcriptomic profile, cancer biomarkers, immunomonitoring, immunosuppression, immunotherapy, myeloid‐derived suppressor cells, pazopanib, renal cell carcinoma, transcriptional modular repertoire analysis, tyrosine kinase inhibitors

## Abstract

**Background:**

The combination of immune checkpoint blockade (ICB) with standard therapies is becoming a common approach for overcoming resistance to cancer immunotherapy in most human malignancies including metastatic renal cell carcinoma (mRCC). In this regard, insights into the immunomodulatory properties of antiangiogenic agents may help designing multidrug schedules based on specific immune synergisms.

**Methods:**

We used orthogonal transcriptomic and phenotyping platforms combined with functional analytic pipelines to elucidate the immunomodulatory effect of the antiangiogenic agent pazopanib in mRCC patients. Nine patients were studied longitudinally over a period of 6 months. We also analyzed transcriptional data from The Cancer Genome Atlas (TCGA) RCC cohort (N = 571) to assess the prognostic implications of our findings. The effect of pazopanib was assessed in vitro on NK cells and T cells. Additionally, myeloid‐derived suppressor (MDSC)‐like cells were generated from CD14^+^ monocytes transfected with mimics of miRNAs associated with MDSC function in the presence or absence of pazopanib.

**Results:**

Pazopanib administration caused a rapid and dramatic reshaping in terms of frequency and transcriptional activity of multiple blood immune cell subsets, with a downsizing of MDSC and regulatory T cells in favor of a strong enhancement in PD‐1 expressing cytotoxic T and Natural Killer effectors. These changes were paired with an increase of the expression of transcripts reflecting activation of immune‐effector functions. This immunomodulation was marked but transient, peaking at the third month of treatment. Moreover, the intratumoral expression level of a MDSC signature (MDSC INT) was strongly associated with poor prognosis in RCC patients. In vitro experiments indicate that the observed immunomodulation might be due to an inhibitory effect on MDSC‐mediated suppression, rather than a direct effect on NK and T cells.

**Conclusions:**

The marked but transient nature of this immunomodulation, peaking at the third month of treatment, provides the rationale for the use of antiangiogenics as a preconditioning strategy to improve the efficacy of ICB.

## Graphical abstract

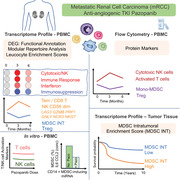

**Abbreviations**
DC: dendritic cell
G‐MDSC: granulocytic myeloid derived suppressor cells
ICB: immune checkpoint blockade
iDC: immature dendritic cell
IPA: ingenuity Pathway Analysis
mDC: myeloid dendritic cells
MDSC: myeloid‐derived suppressor cells
M‐MDSC: monocytic myeloid‐derived suppressor cells
RCC: renal cell carcinoma
mRCC: metastatic renal cell carcinoma
NK: natural killer cell
PBMCs: peripheral blood mononuclear cells
PCA: principal component analysis
pDC: plasmacytoid dendritic cells
PHA: proportional hazard assumption
Tcm: central memory T cell
Tgd: T gamma delta cells
Th1 cells: T helper 1 cells
Th2 cells: T helper 2 cells
TKI: tyrosine‐kinase inhibitor
Treg: regulatory T cell
VEGF: vascular endothelial growth factor

## 1 INTRODUCTION

The immunomodulatory properties of cancer therapies are recently gaining attention in view of potential combinations with immunotherapy such as immune checkpoint blockade (ICB). The nowadays consolidated evidence that ICB mediates effective tumor control only in a minority of patients and in selected malignancies, points to the use of drug combinations as a strategy to increment ICB effectiveness.^1^ Initial results showing increased efficacy of PD‐1 blockers combined with chemotherapy in non‐small cell lung cancer (NSCLC)^2^ and breast cancer,^3^, ^4^ along with multiple clinical trials ongoing in different tumor types, suggest that combinatorial approaches hold promise to become the gold standard of treatment in different settings.

However, the effects of cancer therapies on the multiple components of tumor immunity might be variegated. This complexity needs to be carefully considered when combination strategies based on desired synergies are designed. Antineoplastic treatments might directly potentiate tumor immunogenicity by broadening antigenic repertoire or favoring antigen presentation that boost T‐cell priming^2^; or they can act indirectly by reducing myeloid‐derived suppressor cell (MDSC)‐mediated immunosuppression as a beneficial outcome of their common myelotoxicity.^5^ Conversely, according to in vitro and/or in vivo experimental studies, antiproliferative therapeutic strategies, particularly those based on the inhibition of multiple tyrosine kinases, might affect T‐cell proliferation and function as well, thus, potentially interfering with the protective activity of adaptive immunity.^6^, ^7^, ^8^, ^9^ Hence, gaining detailed information on the type and kinetics of the immunomodulating properties of anticancer drugs would be essential to maximize clinical efficacy when diverse therapeutic strategies are combined with immunotherapy.

The study of tumor immunity is commonly focused on tissue sampling to quantitatively and qualitatively characterize immune cell infiltrate at the tumor site. Nonetheless, tumor lesions are not always accessible (such as in metastatic cancers with visceral metastases), they might be heterogeneous within the same patient and often, because of biopsy related risks, cannot be repeatedly assessed for longitudinal immunomonitoring. Given these limitations, blood‐based analyses, usually not restrained in terms of sampling frequency or accessibility, have been proposed. Moreover, given the systemic nature of tumor immunity,^10^ blood assessments allow to intercept changes in defined circulating immune cells and, hence, provide a more global view of drug‐mediated immunoconditioning.

The complexity of the human immune system and its dynamic nature are moving immunomonitoring approaches toward multiplex and “omics” strategies, with first results emerging in autoimmunity and viral infections.^11^, ^12^ Transcriptomic analysis of peripheral blood,^13^, ^14^ for instance, has been extensively used to dissect mechanisms of action of vaccination against infectious diseases,^15^ to elucidate pathogenic mechanisms of different immunologic disorders,^16^, ^17^ and to identify perturbations associated with different viral,^18^ parasitic,^19^ or bacterial infections^16^, ^20^ including COVID‐19.^21^, ^22^ However, such an approach remains relatively unexplored in the context of cancer therapy including immunotherapy.^23^ Pioneering studies in cancer patients treated with IL‐2 have contributed to the characterization of systemic changes induced by this cytokine.^24^, ^25^, ^26^ More recently, peripheral blood transcriptomic analysis has been used to identify signatures associated with responsiveness to anti‐CTLA4,^27^ and to describe changes differentially associated with CTLA4 and combined CTLA4/PD‐1 blockade.^23^, ^28^ However, no combined transcriptional‐phenotypic analyses to validate whether transcriptomic studies do reflect actual changes of immune cell marker expression and functional features has been performed so far in this setting.

In the present work, we applied an integrative analysis encompassing transcriptional profiling (leucocyte subtype abundance estimation, functional characterization by pathway analysis, and modular repertoire analysis) and multiplex flow cytometry, to comprehensively capture the immunomodulation mediated by antiangiogenic treatment in metastatic clear cell renal cell carcinoma (RCC) patients. Moreover, we also assessed the expression level of the MDSC signatures in The Cancer Genome Atlas (TCGA) clear cell RCC cohort (KIRC) for evaluating prognostic value of these novel MDSC signatures. The peripheral blood analysis was performed before and at different time points during administration of the antiangiogenic drug pazopanib, a tyrosine kinase inhibitor (TKI) included in the standard care of this disease.^29^, ^30^, ^31^ Clear cell RCC, the most frequent RCC histotype, was specifically chosen for (1) the high but partial sensitivity to ICB, leaving up to 50‐60% of patients not responding to such treatment,^32^, ^33^, ^34^ and (2) the potent immunosuppressive properties, linked to hypoxia/VHL‐related oncogenic pathways that lead to the secretion of proangiogenic factors known to mediate the blunting of adaptive antitumor immunity.^35^ In such a scenario, antiangiogenics could be potentially instrumental to the goal of rescuing resistant patients through the downmodulation of immunosuppression and consequently creating a more favorable environment to the stimulatory activity of ICB. We here performed an independent and blinded immunophenotypic and transcriptional analysis of peripheral blood mononuclear cells (PBMCs) obtained from metastatic clear cell RCC (mRCC) patients receiving pazopanib. Our data demonstrates that this antiangiogenic agent mediates a potent but transient reprogramming of systemic immunity, resulting in a contraction of the myeloid suppressor compartment accompanied by an enhanced T‐ and natural killer (NK)‐cytotoxic response. Moreover, the intratumoral expression level of a MDSC signature developed here was strongly associated with poor prognosis outcome in mRCC patients.

Our study indicates that monitoring systemic immunity by transcriptomics may help the designing of combination strategies that could ameliorate clinical efficacy through the timely engagement of drug‐specific immunomodulating properties.

## 2 RESULTS

### 2.1 Integrative transcriptional analysis reveals the immune modulatory properties of pazopanib

The study has been conducted on mRCC patients treated with first‐line pazopanib, whose PBMCs were obtained from blood withdrawn at baseline, 3‐ and 6‐months during treatment. Transcriptional profiling was analyzed using integrative and complementary pipelines.

**FIGURE 1:**
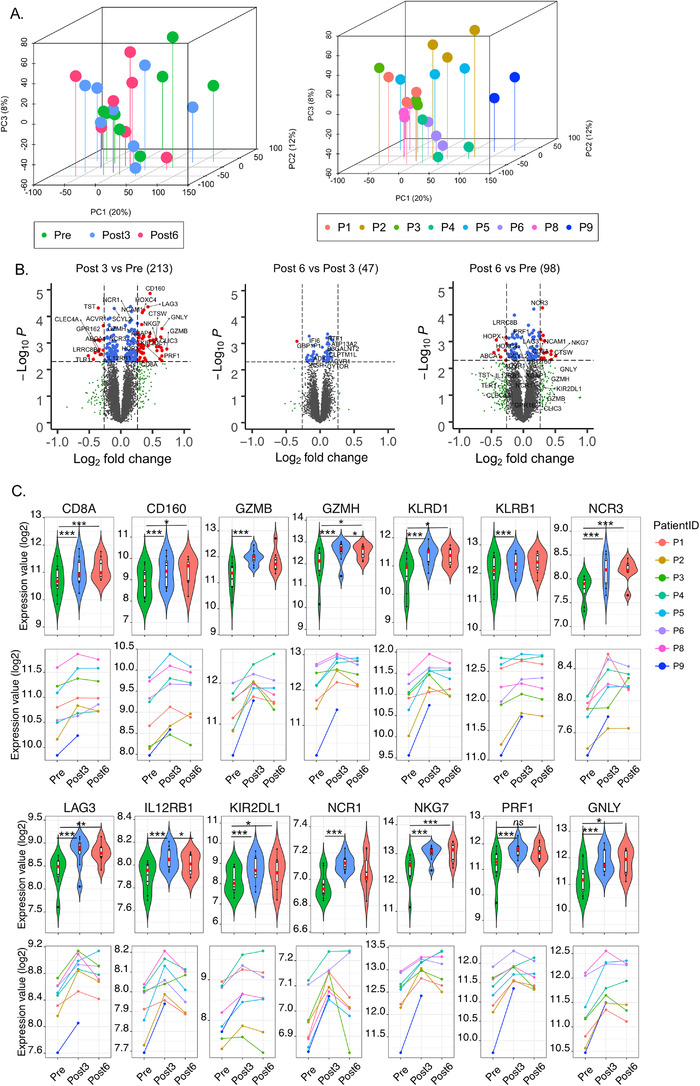
Transcriptional analysis of PBMCs from mRCC patients treated with pazopanib. (A) Principle component analysis (PCA) of all patient samples color coded by time of treatment (left) and individual patient (right). (B) Volcano plots of differentially expressed genes between pre‐ and posttreatment (*Post 3* vs *Pre*, *Post 6* vs *Post 3* and *Post 6* vs *Pre*); the horizontal line represents cut off at *p <* .005 and vertical lines represent fold change >1.2 (right) or <−1.2 (left). (C) Violin and line plots of selected genes. The paired *t‐test* was used for comparison of the expression levels of each gene between patient groups. * represent *p *< .05, ** represent *p *< .01, ***represent *p *<.005

We first wanted to explore the molecular heterogeneity of the sample set through principal component analysis (PCA) based on the whole transcriptomic profile (12 913 genes) (Figure 1A). The first three major PCs accounted for 20.7% (PC1), 10.7% (PC2), and 8.0% (PC3) of the variability observed for these conditions. These three‐dimensional plots showed the distribution of individual patient samples for each time point with no outlier sample (Figure 1A). As expected, a certain degree of separation according to patient ID was observed, which was overall dominant as compared to time‐point effect.

We then performed differential expression analysis between post‐treatment versus pretreatment samples, in which each subject serves as its own comparator, therefore, controlling for interpatients baseline variability. Two hundred and thirteen transcripts were significantly different between 3‐month post‐treatment (*Post 3*) and pretreatment (*Pre*), 47 transcripts between 6 and 3 months post‐treatment (*Post 6 vs. Post 3*), and 98 transcripts between *Post 6* and *Pre*. Volcano plots showing log2 fold‐change (log2FC) and *P‐value (paired t‐test)* of differentially expressed genes are shown in Figure 1B, and Supporting information Table S1. Strikingly, among the top 40 genes ranked according to the log2FC, the large majority was associated with cytotoxic functions and interferon signaling (Table 1). Representative transcripts related to T‐ and NK‐cells cytotoxic functions and T‐cells activation (eg, *CD8A, CD160, GZMB, GZMH, KLRD1, KLRB1, NRC3, LAG3, IL12RB1, KIR2DL1*, *NCR1, NKG7, PRF1*, and *GNLY*) are represented in Figure 1C. The overexpression of such transcripts was attenuated at 6 months after treatment. The common transcripts were significantly upregulated at both *Post 3* and *Post 6 (N = 16)* as compared to pretreatment and include CD8A, *CTSW, NCAM1 (CD56), NCR3, NKG7*, consistent with a persistent but attenuated NK‐ and T‐cell response^36^ (Figure 1B and Supporting information Table S1).

**TABLE 1:** Top 40 of differentially expressed between post‐treatment 3 months versus pretreatment

Symbol	Gene name	*p‐value*	log2FC
GZMB	granzyme B	.00169	0.726
KLRD1	killer cell lectin like receptor D1	.00192	0.651
GNLY	granulysin	.00029	0.649
KLRF1	killer cell lectin like receptor F1	.00308	0.648
PRF1	perforin 1	.00461	0.646
CLIC3	chloride intracellular channel 3	.00150	0.637
KIR2DL3	killer cell immunoglobulin like receptor, two Ig domains and long cytoplasmic tail 1	.00060	0.630
GZMH	granzyme H	.00394	0.616
NKG7	natural killer cell granule protein 7	.00137	0.601
KIR2DL4	killer cell immunoglobulin like receptor, two Ig domains and long cytoplasmic tail 4	.00194	0.570
CST7	cystatin F	.00140	0.564
CTSW	cathepsin W	.00072	0.556
KIR2DL1	killer cell immunoglobulin like receptor, two Ig domains and long cytoplasmic tail 1	.00097	0.525
KIR3DL1	killer cell immunoglobulin like receptor, three Ig domains and long cytoplasmic tail 1	.00077	0.507
CD160	CD160 molecule	.00001	0.461
KIR3DL2	killer cell immunoglobulin like receptor, three Ig domains and long cytoplasmic tail 2	.00044	0.461
RNF165	ring finger protein 165	.00081	0.458
TTC38	tetratricopeptide repeat domain 38	.00058	0.447
KIR2DS5	killer cell immunoglobulin like receptor, two Ig domains and long cytoplasmic tail 3	.00126	0.431
LAG3	lymphocyte activating 3	.00004	0.427
PYHIN1	pyrin and HIN domain family member 1	.00450	0.394
SPON2	spondin 2	.00374	0.377
HOXC4	homeobox C4	.00006	0.363
PTGDR	prostaglandin D2 receptor	.00059	0.362
KLRC1	killer cell lectin like receptor C1	.00128	0.361
IL18RAP	interleukin 18 receptor accessory protein	.00240	0.358
AGAP1	ArfGAP with GTPase domain, ankyrin repeat and PH domain 1	.00055	0.355
NCR3	natural cytotoxicity triggering receptor 3	.00160	0.354
CBLB	Cbl proto‐oncogene B	.00497	0.348
NCAM1	neural cell adhesion molecule 1	.00007	0.339
TSEN54	tRNA splicing endonuclease subunit 54	.00068	0.339
SDF2L1	stromal cell derived factor 2 like 1	.00118	0.339
PDZD4	PDZ domain containing 4	.00247	0.338
PRSS30P	serine protease 30, pseudogene	.00237	0.334
NMUR1	neuromedin U receptor 1	.00020	0.329
IFITM1	interferon induced transmembrane protein 1	.00223	0.328
FKBP11	FK506 binding protein 11	.00128	0.319
KIR3DL3	killer cell immunoglobulin like receptor, three Ig domains and long cytoplasmic tail 3	.00152	0.318
HOPX	HOP homeobox	.00217	0.310
CD8A	CD8a molecule	.00348	0.304

### 2.2 Functional interpretation of transcriptomic changes induced by pazopanib

**FIGURE 2:**
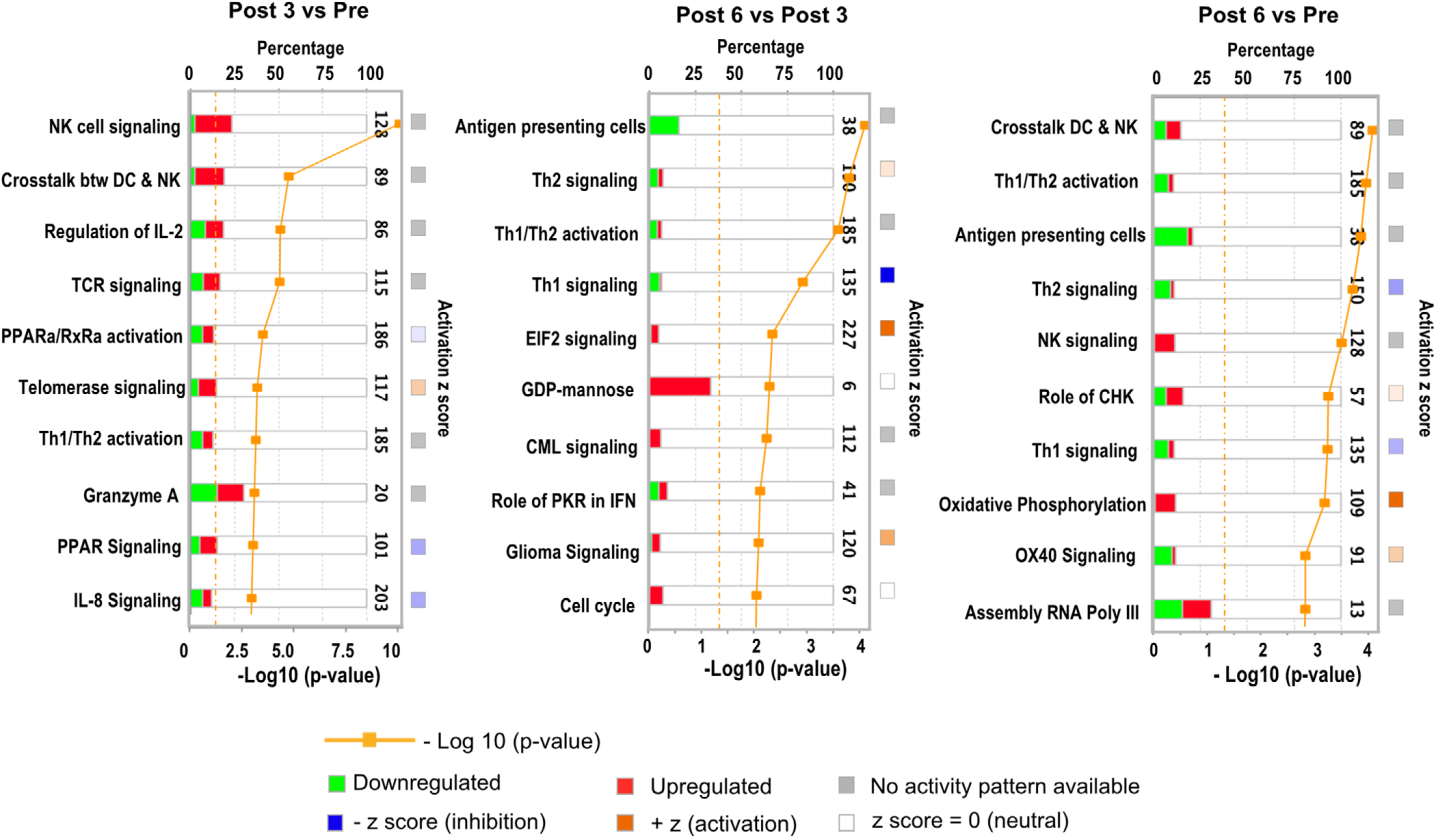
Impact of pazopanib treatment on blood transcriptome (PBMCs) in mRCC patients. Top ten canonical pathways ranking modulated by treatment identified using IPA analysis according to significance level (*paired t‐test, p < *.05). Post 3 versus Pre (left panel), Post 6 versus Post 3 (middle panel), and Post 6 versus Pre (right panel)

The top ten differentially modulated canonical pathways in post‐treatment versus pretreatment samples are shown in Figure 1. The graphical representation of the top pathway at each time‐point comparison is shown in Supporting information Figure S1. The majority of the top canonical pathways modulated by pazopanib (7/10 in both *Post 3* and *Post 6* comparison) were associated with immune functions (Figure 2). The perturbations induced at the third month of treatment are consistent with the triggering of NK/cytotoxic signaling, the positive modulation of the crosstalk between dendritic cells (DCs) and NK cells, the regulation of IL‐2, T‐cell receptor (TCR) signaling, and IL‐8 signaling. After 3 further months of treatment, an attenuation of the immune modulatory effect induced by pazopanib was observed. This was substantiated by the downregulation of transcripts associated with T helper (Th)‐1 and Th2 functional orientation when comparing *Post 6 versus Post 3* samples. The activation of NK‐related pathways was still sustained at the sixth month of treatment, although attenuated.

### 2.3 Pazopanib‐induced molecular fingerprints

**FIGURE 3:**
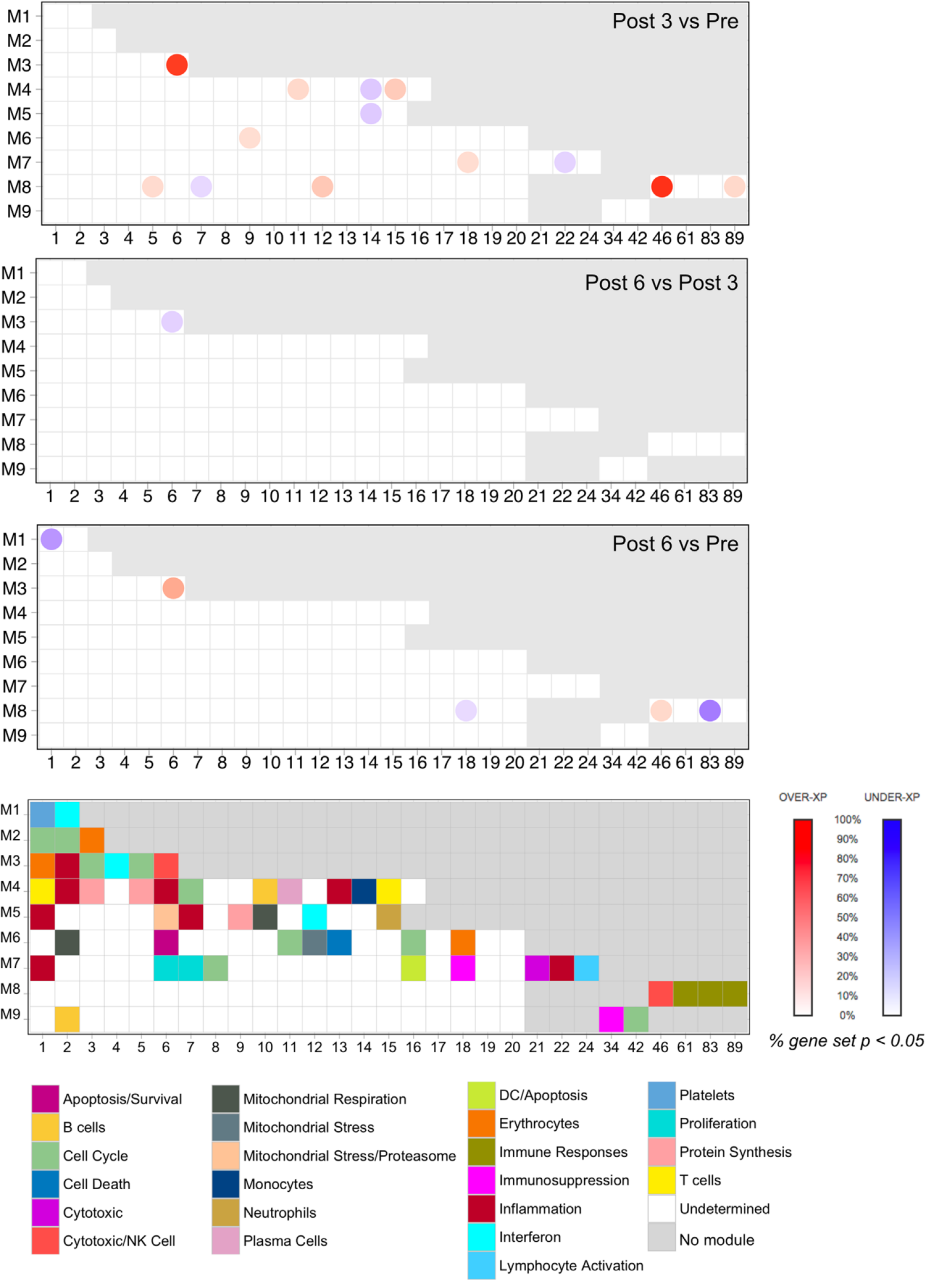
Modular mapping of changes in blood transcriptome (PBMCs) elicited by pazopanib in mRCC patients. Changes in transcript abundance measured in PBMCs using whole‐genome arrays were mapped against a preconstructed repertoire of coexpressed gene sets (modules). The proportion of transcripts for which abundance was significantly changed in comparison between samples collected at 3 months (*Post 3*) versus baseline (*Pre*), 6 months (*Post 6*) versus 3 months (*Post 3*) and 6 months (*Post 6*) versus baseline (*Pre*) in each module. When the percentage of response exceeds 15%, the module was considered as responsive to treatment. Responsive modules are mapped on a grid, the proportion of significant transcripts for each module is represented by a spot of color, with red representing increased abundance and blue representing decreased abundance. The degree of intensity of the spots denotes the percentage of transcripts in a given module showing significant difference in abundance in comparison to the baseline. A legend is provided with functional interpretations indicated at each position of the grid by a color code. Functional interpretations are indicated by the color‐coded grid at the bottom of the figure

**FIGURE 4:**
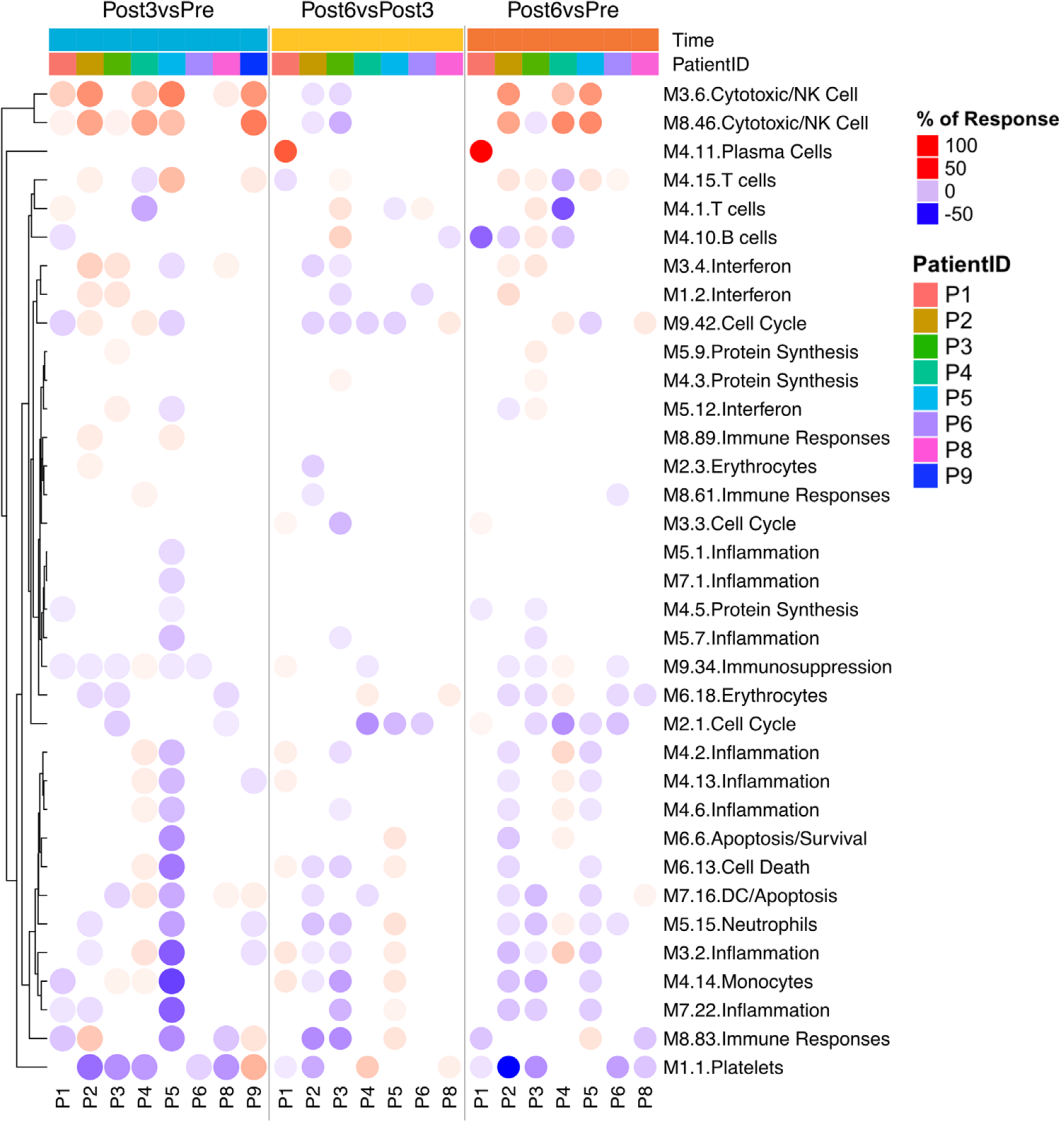
Pazopanib‐induced Perturbations of the modular repertoire across individual samples (PBMCs) of mRCC patients. Percentage of response of individual patients at *Post 3* versus *Pre* (blue), *Post 6* months versus *Post 3* months (yellow), and *Post 6* months versus *Pre* (orange). The expression profile for each individual sample was calculated as a FC and difference relative to an expression of individual samples at each time point. To determine posttreatment changes for individual subjects, a cut‐off is set against which individual genes constitutive of a module are tested (|FC| > 1 and |difference| > 10)

We applied modular repertoire analysis to further dissect the immunomodulatory properties of pazopanib.^13^, ^15^, ^19^, ^37^ ^,^ The percentage of responsive transcripts constitutive of a given module was determined at each time point (see Materials and Methods for details). The group comparison analysis confirmed that module perturbations peaked at 3 months of treatment and decreased at 6 months. These perturbations include the upregulation of modules M3.6 and M8.46 defining cytotoxic/NK cells, M4.11 (plasma cells), and M8.89 (immune response). Moreover, the responsiveness of M4.14 (monocytes) was decreased (Figure 3). However, mapping perturbations of the modular repertoire for a group of subjects does not account for the heterogeneity observed at the individual level. We, therefore, performed deeper individual‐level analysis. This approach demonstrated that pazopanib administration was associated with the decrease of M9.34 (immunosuppressive) in the majority of patients. The most coherent changes were represented by modulations of cytotoxic/NK cells modules (M3.6 and M8.46) while the majority of the other modules demonstrated a considerable heterogeneity. Interestingly, a rapid increase of IFN modules (M1.2 and M3.4) was observed exclusively in patients displaying upregulation of cytotoxic/NK cells modules (Figure 4, Supporting information Table S2).

### 2.4 Modulation of leucocyte functional orientation induced by pazopanib as derived by transcriptomic data

**FIGURE 5:**
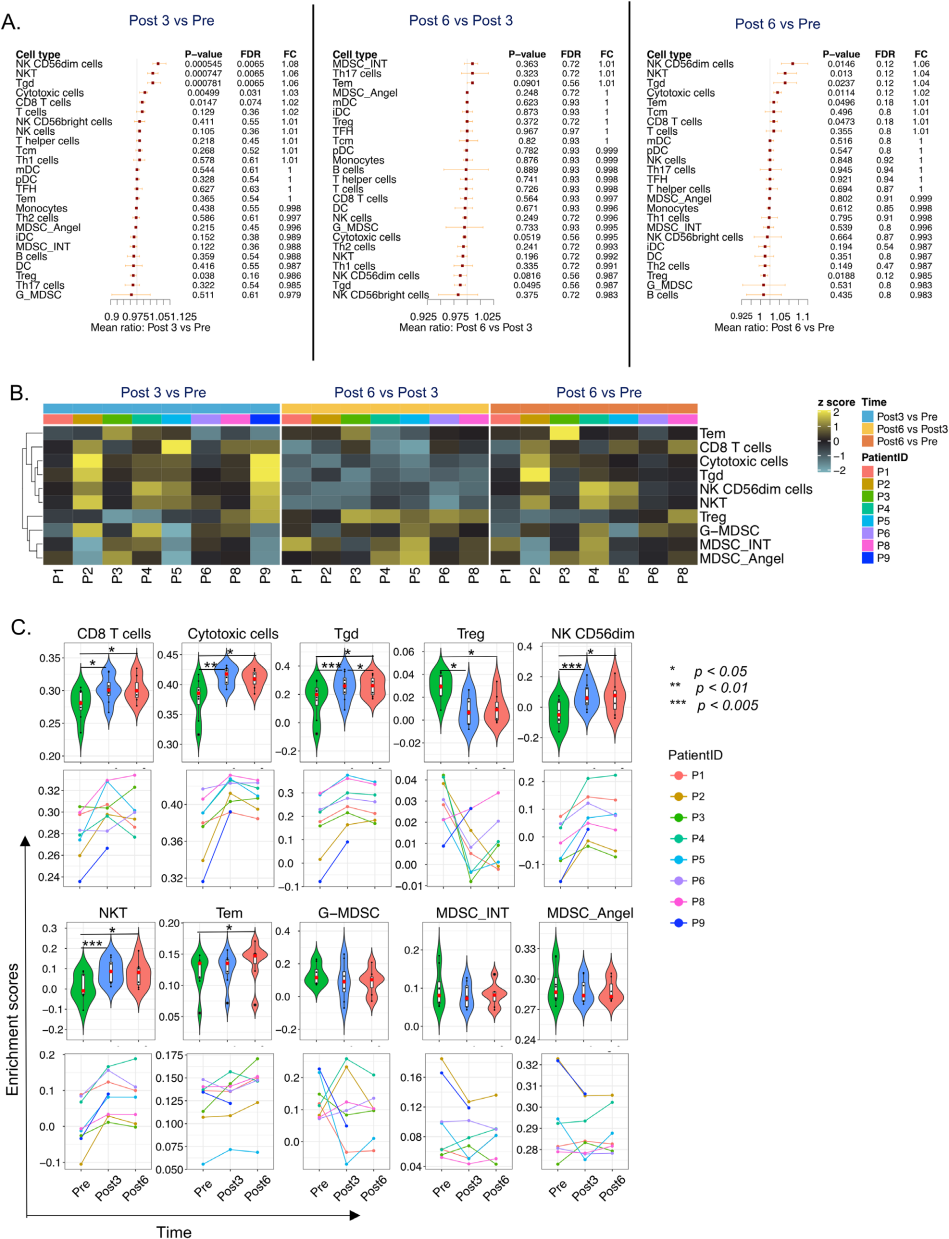
Cell‐type specific analysis in pretreatment and post‐treatment samples. (A) Forest plot of leucocyte enrichment score comparison between *Post 3* versus *Pre*, *Post 6* versus *Post 3*, and *Post 6* versus *Pre*. (B) Heatmap analysis of fold change of leucocyte enrichment score; the fold change‐scored values of representative fold change between *Post 3* versus *Pre*, *Post 6* versus *Post 3*, and *Post 6* versus *Pre* are displayed in a heatmap. (C) Violin plots and line charts of significant cell types. Asterisks: * represent *p *< .05, **represent *p *< .01, *** represent *p *< .005

To estimate the changes in leucocyte populations, we compared enrichment scores generated by single sample Gene Set Enrichment Analysis (ssGSEA). The comparison of post‐treatment versus baseline enrichment scores showed that NKCD56^dim^, T gamma‐delta (Tgd), NKT, cytotoxic cells, and CD8 T cells increased significantly and coherently at 3 months of treatment and subsequently slightly decreased without reaching baseline levels (Figure 5). Conversely, regulatory T cells (Tregs) were significantly downmodulated at the 3‐month time point. A similar trend was observed for MDSCs (Figure 5C; summarized by using three different signatures, see Materials and Methods) with the highest coherence being observed for MDSC_INT. These results suggest that pazopanib induces synergistic immune modulations by enhancing protective immunity and reducing suppressive mechanisms.

### 2.5 Flow cytometry analysis confirms the positive immune modulation associated with pazopanib administration

Transcriptome profiling in bulk cell populations provided a high‐level and unbiased perspective on the changes taking place following initiation of therapy. It is ideally complemented by flow cytometry analyses which provide a targeted but highly granular view of changes taking place at the cellular and protein levels.

**FIGURE 6:**
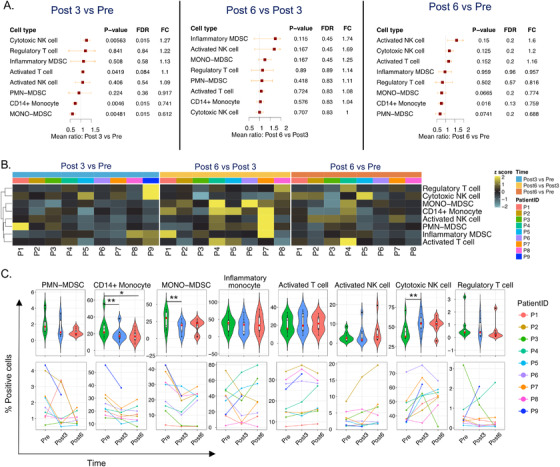
Flow cytometry analysis in samples with pretreatment and post‐treatment. (A) Forest plot of the ratio of cell‐type proportions between *Post 3* versus *Pre*, *Post 6* versus *Post 3*, and *Post 6* versus *Pre* analyzed by flow cytometry. (B) Heatmap analysis of fold change of cell‐type proportions; the *z*‐scored values of representative fold change between *Post 3* versus *Pre*, *Post 6* versus *Post 3*, and *Post 6* versus *Pre* are displayed in a heatmap. (C) Violin plots and line chart of significant cell types. PMN‐MDSC, % CD15^+^ in PBMC (debris exclusion gate); CD14^+^ monocytes, % CD14^+^ in PBMC (debris exclusion gate); MONO‐MDSC, % CD14^+^HLA‐DR^neg^ in CD14^+^ cells; inflammatory monocytes, % CD14^+^PD‐L1^+^ in CD14^+^ cells; activated T cells, %CD3^+^PD‐1^+^ in CD3^+^ T cells; activated NK cells, %CD3^−^CD16^+^CD56^+^PD‐1^+^ in CD3^−^CD16^+^CD56^+^ NK cells; cytotoxic NK cells, % CD3^−^CD16^+^CD56^dim^ in CD3^−^ cells; Treg, % CD4^+^CD25^high^Foxp3^+^ in CD4^+^ cells. Asterisks: * represent *p *< .05, ** represent *p *< .01, *** represent *p *< .005

Multicolor flow cytometry analysis of PBMCs was performed concomitantly in biological replicates of the same blood samples submitted to transcriptional profiling, plus an additional patient for whom RNA was not available. A broad panel of markers encompassing innate and adaptive immune cell subsets of the lymphoid and myeloid repertoire was studied and modulation in on‐treatment with respect to baseline samples was assessed. The analysis showed that pazopanib administration was associated with a remarkable increase of activated and cytolytic effectors, including the subset of activated T cells (CD3^+^PD‐1^+^), reported to contain tumor‐specific T cells,^38^ activated NK cells (CD3^−^CD16^+^CD56^+^PD‐1^+^), and cytotoxic NK cells (CD3^−^CD16^+^CD56^dim^) (Figure 6).^39^ Of note, this evidence is in line with the data that emerged from the transcriptional profiling, depicting an overall boost of genes involved in TCR signaling, cytotoxic cell populations, and NK activity. Again, similarly to findings obtained via transcriptomic analyses, the detected changes over baseline were more evident at 3 months of therapy and tended to a plateau or decreased at 6 months. The boost of T‐ and NK‐cell activation was paralleled by a significant decrease in the frequency of different myeloid cell subsets including CD14^+^ monocytes and monocytic (MONO‐)MDSCs (CD14^+^HLA‐DR^neg^)^40^ (Figure 6). Inflammatory monocytes (CD14^+^PDL‐1^+^),^41^ PMN‐MDSCs (CD15^+^),^40^ and Tregs (CD4^+^CD25^high^Foxp3^+^) also displayed a remarkable reduction during treatment in the majority of patients. Changes of all these cell subsets were mostly detectable at 3 months during treatment with respect to baseline, with a stabilization of monoytic MDSCs or/and a rebound for total and inflammatory monocytes in cell frequencies at 6 months (Figure 6). Taken together, the kinetics of immune modulation as detected by flow cytometry are in line with those emerging from the transcriptional profiling data and confirm the transient nature of immunomodulation mediated by pazopanib.

### 2.6 Pazopanib decreases MDSC‐mediated immune suppression in vitro

**FIGURE 7:**
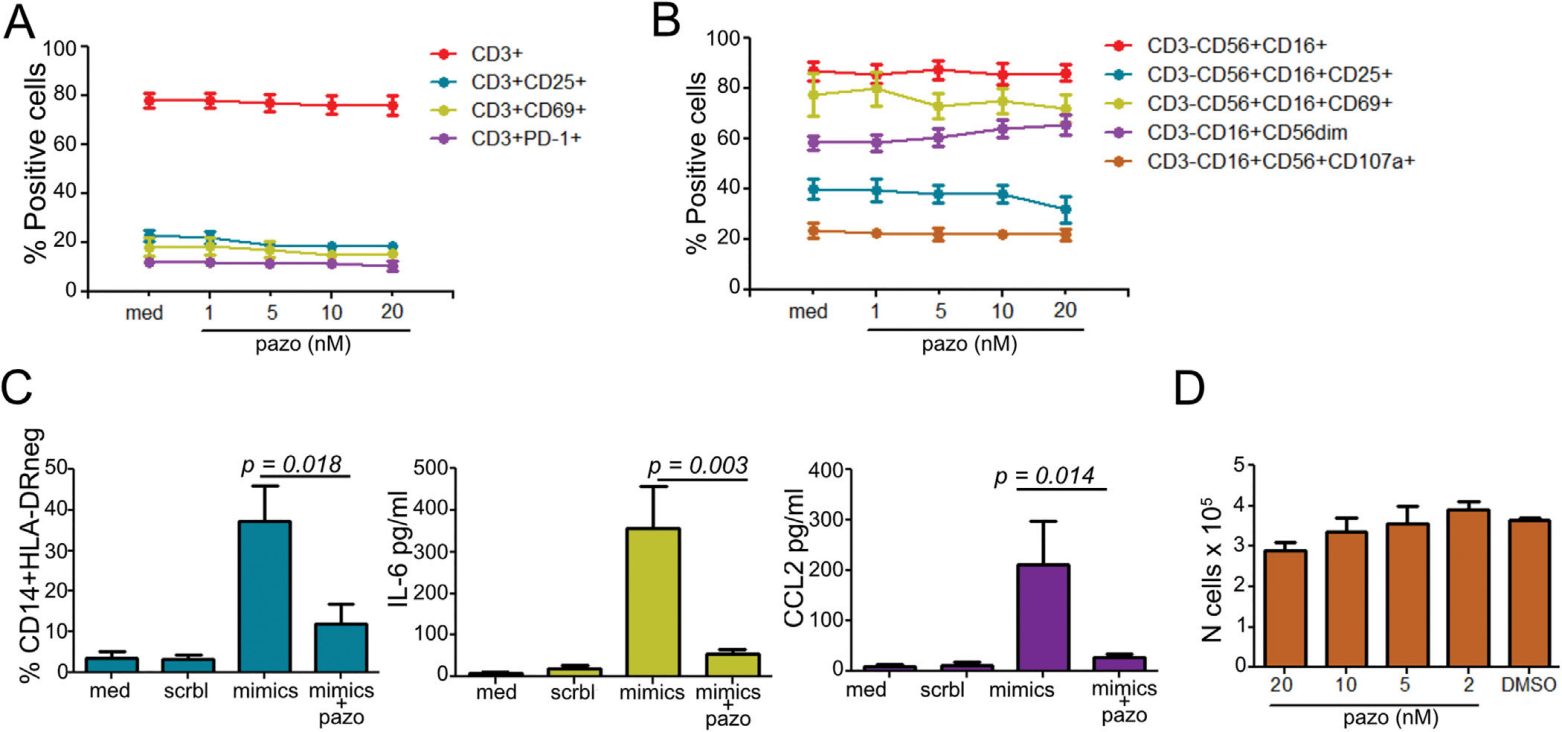
Effect of Pazopanib on immune cell subsets in vitro. (A) CD14‐depleted PBMCs from healthy donors (n = 3), activated with CD3/CD28 beads were cultured for 3 days in medium (med) additioned or not with different concentrations of pazopanib (nM) reflecting the in vivo drug level ranges. Cells gated on CD3^+^, were profiled by flow cytometry for the indicated markers. (B) CD14‐ and CD3‐depleted PBMCs were cultured for 5 days with IL‐2 and IL‐12 with or without pazopanib (nM). Cells were profiled by flow cytometry as indicated and gated as CD3^−^CD56^+^CD16^+^ cells. (C) CD14^+^ cells were treated overnight with a MDSC‐like cell inducing miRNA cocktail (mimics) scramble controls (scrbl), or medium (med), prior to pazopanib treatment (5 nM; mimics + pazo) for 24 h. Cells and supernatants were evaluated by flow cytometry and cytokine bead array array (CBA). (D) CD14^+^ cells were incubated overnight with pazopanib (nM) and analyzed for cell viability by trypan blue exclusion. In all experiments, DMSO was used as the highest pazopanib concentration (20 nM). Paired *t*‐test:* represent *p *< .05, ** represent *p *< .01

The boost in T‐ and NK‐cell activation and cytolytic functions observed by both transcriptomics and flow cytometry prompted us to investigate whether pazopanib has a direct activity on these immune cell subsets. PBMCs of three healthy subjects were treated with different doses of pazopanib and then analyzed by flow cytometry. The result showed that, in both CD3^+^ T cells and NK cells, pazopanib did not mediate any significant impact on cell activation and cytolytic potential (Figure 7A and B). Only a negligible increase of the CD3^+^CD56^dim^CD16^+^ cells within the NK cell culture was observed with increasing doses of pazopanib, while the other markers were instead stable in expression or decreased (Figure 7B). In contrast, by applying the in vitro MDSC model recently defined by our lab and consisting in the in vitro transfection of a MDSC‐related miRNA panel,^42^ a significant decrease in the frequency of CD14^+^HLA‐DR^neg^ cells, as well as in the secretion of IL‐6 and CCL2, was observed when cells were concomitantly treated with pazopanib at 5 nM, indicating a potential inhibitory effect of the drug on monocyte conversion into MDSC (Figure 7C). In addition, a 20% decrease in viability could be detected at the highest pazopanib concentration (Figure 7D). Altogether, these data indicate that the modulating effect of pazopanib on T‐ and NK‐cell activity observed in treated patients is more likely associated with an indirect relief of cytotoxic functions mediated by the inhibition of MDSC‐mediated immune suppression rather than a direct effect on NK‐ and T‐effector populations.

### 2.7 Intratumoral estimates of MDSC are associated with poor prognosis in kidney cancer

One of the more remarkable findings obtained through combined transcriptomic and flow cytometry‐based immune monitoring is the decrease in MDSC populations and associated signatures.

**FIGURE 8:**
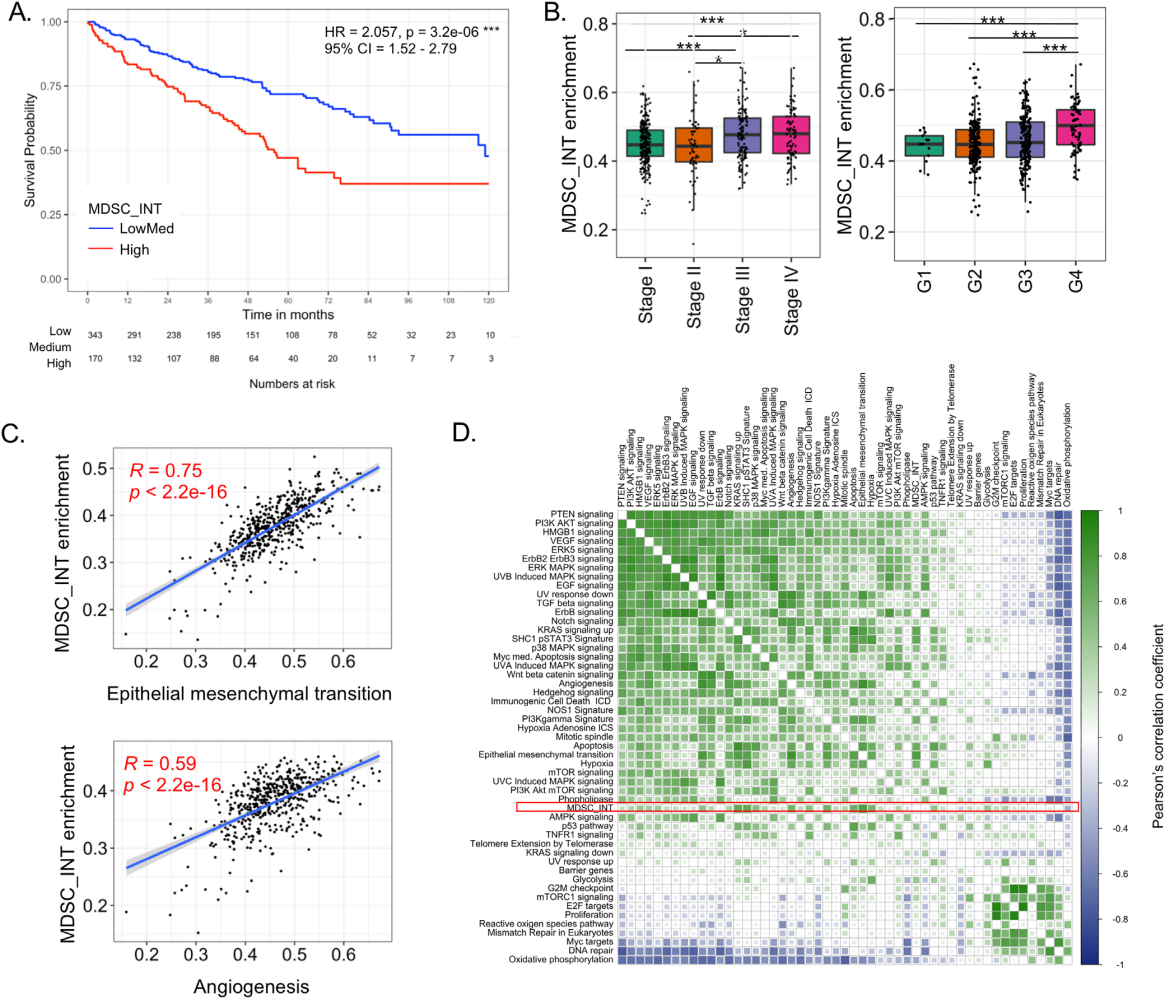
Prognostic implications of MDSC gene signature in TCGA clear‐cell renal cell carcinoma (KIRC, n = 515). (A) Kaplan–Meier curves showing overall survival (OS) of patients within the highest tertile of MDSC_INT enrichment versus the two lower tertiles (LowMed). Cox proportional hazards statistic are shown. (B) Boxplots of MDSC_INT enrichment scores by AJCC pathologic stage (left) and histological grade (right). *t*‐test: * represent *p *< .05, ** represent *p *< .01, *** represent *p *< .005. (C) Scatterplots showing the association between MDSC_INT scores and the enrichment score (ES) of genes related to epithelial mesenchymal transition (upper), and angiogenesis (lower). Regression line with corresponding Pearson's correlation coefficient (R) and *p*‐value are shown. (D) Pearson correlation matrix of enrichment scores of tumor‐associated pathways. MDSC_INT signature is indicated with a red square.

To explore the relevance of our observation, and as no data exist regarding the prognostic role of MDSCs in kidney cancer, we assessed the expression of the three MDSC signatures in TCGA clear cell RCC cohort (KIRC, N = 517, Figure 8 and Supporting information Figure S2). The MDSC_INT signature was strongly associated with decreased overall survival (OS) (MDSC_INT High vs LowMed, hazard ratio (HR) = 2.057 (95% CI = 1.52‐2.79, Figure 8A). In particular, the MDSC High group had poor prognosis, while the MDSC Low and Med groups (Supporting information Figure S2) have similar favorable prognosis. No such differences were observed using the other two MDSC signatures MDSC_Angel and granulocytic myeloid‐derived suppressor cell (G‐MDSC), suggesting that MDSC_INT, which was developed experimentally based on extracellular vesicle‐driven monocyte‐MDSC differentiation, might represent a novel prognostic biomarker in kidney cancer. Remarkably, MONO‐MDSCs were strongly suppressed after pazopanib treatment (Figure 6). MDSC_INT correlates with Stage and Grade, which are major prognostic factors in kidney cancer (Figure 8B). We then assessed the relationship between MDSC_INT with the disposition of oncogenic pathways, and found that MDSC_INT expression linearly correlates with many oncogenic processes associated with cancer aggressiveness, including angiogenesis (*R* = 0.59, *p *< 2e‐16), and epithelial‐to‐mesenchymal transition (EMT) (R = 0.75, *p *< 2e‐16, Figure 8C and D) although there was no overlapping between MDSC_INT signature and angiogenesis or EMT (Supporting information Figure S3). Despite the correlation with Stage and Grade, MDSC_INT retained its prognostic value even when included in a Cox regression multivariable model (Table 2).

**TABLE 2:** Univariate and multivariate overall survival cox proportional hazards regression including MDSC_INT enrichment scores and grade, in TCGA clear cell RCC cohort (KIRC, N = 517)

	Univariate		Multivariable		Stratified mutivariable	
Variables	HR (95% CI)	*p value*	HR (95% CI)	*p value*	HR (95% CI)	*p value*
∼ MDSC signature (High vs. LowMed)	2.057 (1.519‐ 2.787)	3.2e‐06	1.478 (1.081‐ 2.019)	.01425	1.443 (1.054‐1.976)	.02205
∼ Stage	2.057 (1.519‐ 2.787)	3.2e‐06	1.478 (1.081‐ 2.019)^#^	.01425	strata	
∼ Histological grade	2.778 (1.953‐ 3.951)	<2e‐16	1.761 (1.213‐ 2.554)	.00289	1.703 (1.172‐ 2.475)	.00523

Signif. codes: *** <.001; ** <.01; * <.05.

^#^ Significant violation of proportional hazards assumption (PHA).

MDSC_INT signature entered as categorical (factor) variable (factor levels: “MDSC High”, “MDSC LowMed”).

HRs (hazard ratios) for death. Stage was categorized as III & IV versus I & II, and histological grade as G3 & G4 versus G1 & G2. The stratified multivariable model includes stage as stratification factor as it was the only variable that did not meet the proportional hazard assumption.

In addition, we observed a positive correlation between NK‐ and T‐cell signatures (ie, NKCD56dim, NKCD56 bright, T reg, Th1 cells, Th2 cells, and CD8 T cells) among each other and with MDSC_INT (Supporting information Figure S2). We have previously observed that in KIRC TCGA,^43^ a cytotoxic/Th1 phenotype (called ICR high) correlates with negative survival, and the same trend, albeit not statistically significant was observed for Tregs (Supporting information Figure S2). Renal cell carcinoma is unique on its own as high Th1/T cytotoxic cells, in addition to Tregs, have been correlated with negative survival, while in most of the solid tumors the reverse is observed for Th1/T cytotoxic cells.^43^, ^44^, ^45^, ^46^, ^47^, ^48^ It might be possible that this phenomenon is partially due to the concomitant presence of MDSC‐related suppressive mechanisms which impair T‐cell function. Supporting this hypothesis is the observation that T‐cell‐related immune signatures do not predict outcome to ICB alone but are strongly predictive when PD‐1 blockade is combined with antiangiogenic therapy.^49^

Thus, the analysis of the tumor specimens from the TCGA cohort permitted to expand our initial finding by providing indirect evidence about the role of MDSCs in mRCC progression. Indeed, this in turn suggests that MDSC suppression by pazopanib may be one of the means by which treatment could contribute to improved outcome.

## 3 DISCUSSION

This is the first translational study that investigates longitudinally the immunomodulatory effects of an antiangiogenic therapy on PBMCs of kidney cancer patients through concomitant transcriptomic and phenotypic analysis. Our results show that pazopanib triggers cytotoxic cells and IFN pathways and relieves immunosuppression by reducing MDSCs. Interestingly, data from our team indicate that cabozantinib also mediates comparable changes in blood immune phenotype,^50^ thus suggesting a common immunological behavior of this TKI drug family. The invigoration of antitumor immunity mediated by pazopanibwas mostly evident after 3 months of therapy and less accentuated at the sixth month of treatment. The transcriptional profiling of PBMCs clearly revealed treatment‐induced immunomodulation, detecting modifications validated by flow cytometry, but also expanding them by revealing pathway networks and broader functional information. Our data indicate that the analysis of transcriptional profiles of blood cells with the support of appropriate deconvolution approaches represents a valid strategy for monitoring immune cell behavior at a high throughput and reliable level.

To achieve the results reported here, one of the major challenges was the identification of gene signatures appropriate to capture the activity of circulating MDSCs. Indeed, monocytic MDSCs are defined in flow cytometry only by the lack/low expression of HLA‐DR in cells expressing the monocytic marker CD14, alone or in combination with CD11b and CD33, but their genomic features have been poorly defined. To this aim, we used the dataset selected by Angelova^51^ and Fridlender^52^ as reference transcriptional data for myeloid cells and the data set obtained from human MDSCs generated in vitro according to a model developed in our laboratory.^42^ This MDSC model was produced by exposing blood CD14^+^ monocytes to tumor extracellular vesicles, a process leading to cells highly overlapping for phenotype, immunosuppressive function, and transcriptional profiles with MDSCs isolated from blood of melanoma patients.^42^, ^53^, ^54^ The gene signature reflects most of the signaling pathways expected for these cells and overlap with monocytes sorted from cancer patients.^42^ Our MDSC signature, applied to bulk tumors, was the only one with prognostic implications in RCC, confirmed in multivariable analyses, providing here an essential contribution for the estimations of MDSCs in different tissues. Notably, high levels of MDSC subsets in the tumor or in the blood, evaluated by flow cytometry, have been recently associated with a trend for shorter survival and with higher tumor grade, respectively, in RCC patients.^55^

Antiangiogenic TKIs, such as pazopanib, are multikinase inhibitors available for the standard‐of‐care first‐line treatment of mRCC patients, particularly for “low risk” cancers according to Heng criteria.^56^ These TKIs are multitarget agents as they inhibit not only the vascular endothelial growth factor receptor (VEGFR), but also platelet‐derived growth factor receptor, KIT (proto‐oncogene receptor tyrosine kinase), fibroblast growth factor receptors (FGFR), and RAF kinases.^29^, ^30^, ^31^ At present, we do not know whether this broad activity is required for multifaceted immunomodulating activity observed in vivo, or if the selective inhibition of the single VEGF pathway, such in the case of the emerging treatment axitinib,^57^ would induce comparable or even superior effects. In this regard, blood transcriptional studies of mRCC patients under diverse antiangiogenic agents should be performed. Once solid markers have been identified, clinical implementation could be performed by exploiting more standardized clinical‐grade platforms such as Nanostring.^58^, ^59^

It is tempting to speculate that the general immunological reshaping observed following pazopanib might stem from the blunting of MDSC and Treg immunosuppression. The effect could be a bystander consequence of the known TKI myelotoxicity,^60^ or instead the result of blocking activity on VEGFR and KIT downstream signaling pathways.^8^, ^49^, ^50^ The reduced myeloid immunosuppression could then consent the relief of T‐ and NK‐cell cytolytic functions broadly observed by both transcriptomics and flow cytometry in treated patients. This hypothesis is corroborated by our in vitro studies showing no major change in the activation of NK and T cells when the drug was provided. Instead, we observed that pazopanib significantly impairs in vitro MDSC conversion from normal monocytes and exerts a moderate reduction of myeloid cell survival upon addition to culture medium. This is in agreement with clinical data showing a certain level of myelotoxicity mediated by pazopanib as well as other antiangiogenic TKIs.^61^ Nevertheless, we could not rule out that more subtle modulating effects, direct or indirect, could occur at transcriptional or functional level in NK and T cells, or take place in vivo in the tumor microenvironment and immune organs. For instance, a recent study proposed that pazopanib induces DC activation by inhibiting the ß‐catenin pathway in vitro,^62^ with possible enhancement of T‐cell responses,^63^ while novel VEGF‐directed drugs, such as axitinib, increase the expression of NKG2D ligands in the tumor cells and consequently potentiate NK cell cytolytic activity.^64^ A recent report in a melanoma patient experiencing kidney allograft rejection following PD‐1 blockade suggests that mTOR inhibitors can increase tolerogenic mechanisms against nontumoral tissue at the same time preserving the activity of checkpoint inhibitors.^65^ This observation, coupled with the recent findings that pazopanib and mTOR inhibitors can be particularly effective in tumors bearing specific genetic alterations (mTOR and FGFR pathway mutations) offer the opportunity to study additional combinatorial approaches in selected patient populations.^66^

To perform our analyses, we collected peripheral blood before and 3‐6 months after treatment intiation. These time points were chosen considering findings and limitations of relevant investigations in this context. Studies in humans have shown that anti‐VEGF TKIs, such as sunitinib, reduce monocytes^67^ (assessed 4 and 6 weeks post‐treatment), monocytic MDSCs^68^ and Tregs^69^ (assessed at 4 weeks after treatment only). Even if perturbations could occur at earlier time points, it might be difficult to translate such findings into clinical practice. In fact, proposing an ultrashort preconditioning treatment (based on extremely transitory immunological effects) might result in suboptimal antitumor activity. Moreover, the reduction of Tregs in peripheral blood after sunitinib administration (assessed after the first, second, and third cycle) was maximal after the third cycle and correlated with prolonged OS.^70^ As sunitinib is administered orally for 4 weeks followed by 2 weeks without treatment (6‐week cycle) while pazopanib is administered continuously, a 3‐month time point was deemed to be ideal to detect clinically relevant changes. Our transcriptomic results show a strong reduction of Treg frequency (Figure 5), which however was not consistently reflected by flow cytometry measurement (Figure 6). This discrepancy may derive from a less precise capturing of Tregs due to the lack of CD127 in the flow cytometry panel, a limitation of our study. On the other hand, this may also indicate that transcriptomics has captured an additional suppressive signature, which is not directly associated with Tregs. This will be object of further investigations.

There are no studies that have assessed immunologic perturbations in patients treated with pazopanib as first‐line treatment. Only one study has assessed immunologic changes in mRCC patients treated with pazopanib, but administered as third‐line treatment, therefore, enrolling patients with a potentially heavily compromised immune system. In their work, Pal et al.^71^ observed that nonresponder patients displayed lower levels of HGF, VEGF, IL‐6, IL‐8, and soluble IL‐2R and increased numbers of monocytic MDSCs as compared with responders at the post‐treatment time point measured at 6 and 12 months after treatment initiation. The fact that Pal et al. could not detect an overall decrease of MDSCs as compared to baseline might depend on late time‐point analysis.^71^ Our study demonstrated that this might be actually the case since perturbations peaked at the third month of treatment and were attenuated after an additional 3‐month exposure, corroborating the rationale of our time‐point selection.

A limitation of our study is that the number of patients analyzed here was rather small. However, it reflected the rarity of mRCC patients who could be prospectively enrolled for first‐line TKI administration especially in research hospitals with competitive clinical trials enrolling. Even so, dynamic changes were extremely coherent across patients and confirmed using orthogonal immune monitoring platforms and analyses, resulting in statistically significant differences.

The data reported here provide a set of key information that might have relevant implications for the design of combinatory treatment strategies in mRCC clinical setting. First, TKIs mediate a specific reshaping of tumor immunity that should favor a prompter response to immunotherapy due to the decrease of immunosuppressive effectors and the concomitant boost of PD‐1^+^ T cells and NK cells. Of note, this precise blood immune scenario has been recently shown to predict response to ICB in NSCLC.^72^ Second, this effect reaches its peak at the third month of treatment but tends to be attenuated at later time points likely due to the homeostatic mechanisms that regulate systemic immunity and tumor‐mediated immunosuppression. These data indicate that a short‐term preconditioning treatment with antiangiogenics might induce a “breach” in systemic immunosuppression and create the optimal immune setting for ICB to potentiate antitumor immune responses in vivo. However, the effect is transitory and would possibly need ICB administration to trigger persistent immune activation and protective memory. Our data suggest that the combination of TKIs with PD‐1 blockers, which might result in unmanageable toxicity,^73^ could be potentially replaced by intermittent schedules, to maximize immunological synergies and possibly improve treatment tolerability. The coadministration of PD‐1/PD‐L1 inhibitors with antiangiogenic monoclonal antibodies (bevacizumab)^74^ or TKIs, such as axitinib, lenvatinib, and cabozantinib,^60^, ^75^, ^76^ is rapidly emerging as a strategy to increase OS in mRCC patients. In this context, our data suggest that more dynamic and innovative approaches based on intermittent or alternate schedules could be also explored to ameliorate the therapeutic index of combinatorial regimens in cancer.^77^, ^78^

## 4 MATERIALS AND METHODS

### 4.1 Patients and study description

From January 2016 to June 2016, nine patients (eight males, one female) with metastatic RCC and clear cell histology (mRCC) were treated with first‐line pazopanib as per clinical practice at the Fondazione IRCCS Istituto Nazionale dei Tumori, Milan, Italy. Safety assessment included physical examination and laboratory tests every month. All patients had a good performance status (ECOG 0:8/9, ECOG 1: 1/9), a median age of 65 years and prevalence of intermediate risk according to Heng score (5/9). They received pazopanib at a standard dose of 800 mg orally once daily, continuously, for at least 6 months. All patients signed an informed consent according to a protocol approved by the INT Ethical Committee [INT146/14]. Pazopanib adverse events (AEs) observed in the present study were in line with literature and with our previous experience.^79^The most common AEs were diarrhea (33%), fatigue (47%), hypertension (7%), mucositis (7%), and skin rash (7%), which are consistent with data reported for other antiangiogenic agents such as for sunitinib and sorafenib. While correlations between toxicity and immune‐related changes were not planned as for study design, no apparent link between toxicity and immune‐related effects was observed, albeit the limited number of patients here analyzed does not allow to reach any definitive conclusion.

### 4.2 Blood collection

Blood samples (30 mL) were obtained from nine patients at baseline (*Pre*), and at the third and sixth month during therapy (*Post 3* and *Post 6*). For one single patient, samples were collected only at baseline and at 3‐month therapy. Blood was processed within 1 h from withdrawal. PBMCs were separated by Ficoll gradient (Leuco‐sep tubes, ThermoFisher Scientific) and viable cells stored in liquid nitrogen until use, or frozen in Qiazol (Qiagen) for RNA extraction and gene expression profiling.

### 4.3 Transcriptomic analysis

Suitable material for transcriptional analysis was available from eight patients. RNA was extracted using miRNeasy kit (Qiagen). After quality check and quantification by 2100 Bioanalyzer system (Agilent) and Nanodrop ND‐1000 spectrophotometer (ThermoFisher), respectively, RNA expression was assessed using Illumina HT12v4 BeadChip. Illumina's BeadStudio version 1.9.0 software was used to generate signal intensity values from the scans. Data were further processed using the Bioconductor “Lumi” package. Following background correction and quantile normalization, expressions were log2‐transformed for further analysis. Raw expression and normalized data matrix have been deposited at NCBI's Gene Expression Omnibus database (http://www.ncbi.nlm.nih.gov/geo/), with accession numbers GSE146163. From a total of 47 323 probes arrayed on the Illumina HT12v4 beadchip, the probes targeting multiple genes were collapsed (average expression intensity) and a final data matrix containing 12 913 unique genes was generated. Data analyses were performed using R (version 1.0.44, RStudio Inc.) and Ingenuity Pathway Analysis (IPA) (QIAGEN Bioinformatics). The comparison between each group (*Post 3 vs pre*, *Post 3 vs Post 6*, and *Post 6 vs Pre*) was performed using paired *t*‐test. For detection of differentially expressed genes, we used a *p* value cutoff of 5 × 10^−3^, and false discovery rate was provided as descriptive statistic (Supporting information Table S1), and not to dictate significance as the risk of type I error was mitigated by the use of orthogonal platforms (ie, flow cytometry) for validation purposes.

Hierarchical clustering was performed using the function “Heatmap” from the R package “ComplexHeatmap.”^80^ Euclidean distance and complete linkage methods were used by default. PCA was performed using the R function “scatterplot3d” package. The first three principal components, PC1, PC2 and PC3, were plotted against each other.

#### 4.3.1 Pathway analysis

Gene ontology analyses were performed using IPA (QIAGEN Bioinformatics). A permissive *P‐*value cut‐off of .05 was used to select transcripts for pathway analysis. The proportion of upregulated and downregulated transcripts was represented. The *z*‐score was used to indicate the direction of pathway deregulation. Transcripts from the top three pathways in each comparison group were plotted in the corresponding heatmaps.

#### 4.3.2 Leucocyte subset estimations

To estimate the enrichment of various cell types, gene expression deconvolution analyses were performed with ssGSEA^81^, ^82^ implemented in the “GSVA package” using cell‐specific signatures (Supporting information Table S3): T cells, CD8 T cells, cytotoxic T cells, T helper 1 cells (Th1 cells), central memory T cells (Tcm), Tem, T‐helper cells, Tfh, Th2 cells, Th17 cells, gamma delta T cells (Tgd), natural killer cells (NK cells), NK CD56^dim^, NK CD56^bright^ cells, B cells,^83^ Treg, NKT cells, DCs, immature DCs (iDC), plasmacytoid DCs, myeloid DCs (mDC).^51^ For MDSCs, we constructed a specific signature based on 25 genes highly correlated in the present dataset, selected from the top 100 genes upregulated in extracellular vesicle‐MDSCs versus monocytes (MDSC_INT, Supporting information Figure S4) in our recent work.^42^ Additional MDSC signatures include the one proposed by Angelova et al.^51^ (MDSC_Angel), based on markers selected according to the literature, and a G‐MDSC signature defined by comparing G‐MDSC versus naïve neutrophils.^52^ Enrichment scores (ES) were calculated by ssGSEA on the log2 transformed data. Forest plots were plotted by using mean 2^ES^ ratio between *Post 3 versus Pre*, *Post 6 versus Post 3*, and *Post 6 versus Pre*. Differentially expressed ES between pretreatment and post‐treatment were calculated through paired *t‐*test (*P* < .05).

#### 4.3.3 Modular repertoire analysis

A set of 260 modules (coexpressed genes) was used for the analysis of this data set. This fixed modular repertoire was a priori determined, being constructed based on coexpression measured across nine reference datasets encompassing a wide range of diseases (infectious, autoimmune, inflammatory)^15^, ^16^, ^37^ (https://github.com/Drinchai/DC_Module_Generation2). This data‐driven approach allowed the capture of a broad repertoire of immune perturbations, which were subsequently subjected to functional interpretation. This collection of annotated modules was then used as a framework for analysis and interpretation of our blood transcriptome dataset. The approach used for the construction, annotation, and reuse of modular blood transcriptome repertoires was previously reported.^12^, ^13^, ^15^, ^16^, ^37^ After normalization, raw expression intensity was used for the module analysis. Briefly, data were transformed from gene‐level data into module (M)‐level activity scores, both for group comparison (*Post 3 vs Pre, Post 6 vs Post 3*, and *Post 6 vs Pre*) and individual patients’ comparison at each time point. The modules defined by this approach (M1‐M9, a total of 260 modules) were used as a framework to analyze and interpret this dataset. For group comparisons, the expression profile at each time point was calculated as a FC relative to a mean expression of all samples within that time points. Then, paired *t‐*test was used to evaluate each time‐point comparison. If the FC between each group comparison was greater than 1, and the *p* value <.05, the transcript was considered as upregulated. If the FC between each group comparison was less than 1, and the *p* value <.05, it was considered as downregulated. Then the percentages of “module responsiveness” were calculated for each module. For individual comparison, the expression profile for each individual patient was calculated as a FC and difference relative to the expression of individual samples at each time point. If the FC between each time point comparison was more than 1, and difference more than 10, the transcript was considered as upregulated. If the FC between each time point comparison was less than 1, and the difference less than 10, it was considered as downregulated. For both, group and individual comparisons, the “module‐level” data are subsequently expressed as a percent value representing the proportion of differentially regulated transcripts for a given module. A module was considered to be responsive when more than 15% of the transcripts were down‐ or upregulated.

### 4.4 Multiparameter flow cytometry

PBMC samples from nine patients were thawed and tested simultaneously for all time points by flow cytometry. Phenotypic profiling was performed after labeling PBMCs with monoclonal fluorochrome‐conjugated antibodies: CD14 FITC (Clone M5E2, BD Pharmingen), CD3 FITC (Clone UCHT1, BD Biosciences), or KO525 (Clone UCHT1, Beckman Coulter), PD‐1 APC (Clone MIH4, BD Pharmingen) or PC7 (Clone PD1.3, Beckman Coulter), HLA‐DR APC (Clone G46‐6, BD Pharmingen), CD15 PerCP‐CY5.5 (Clone HI98, BD Pharmingen), PD‐L1 PE (Clone MIH1, BD Pharmingen), CD4 PE (Clone RPA‐T4, BD Pharmingen), CD25 PerCP‐Cy5.5 (Clone M‐A251, BD Pharmingen), CD56 ALEXA750 (Clone N901, Beckman Coulter), CD16 BV650 (Clone 3G8, BD Biosciences), Live/Dead Fixable Violet (ThermoFisher), FOXP3 APC (Clone FJK‐16s eBioscience) used after cell permeabilization with the kit Perm Buffer (10×) and Fix/Perm Buffer (4×) (BioLegend), according to manufacturer's instructions. Samples were incubated with Fc blocking reagent (Miltenyi Biotec) for 10 min at room temperature before the addition of monoclonal antibodies for 30 min at 4°C. Thereafter, samples were washed, fixed and acquired by Gallios Beckman Coulter FC 500 or BD FACSCalibur (BD Biosciences) flow cytometers, and analyzed with Kaluza software (Beckman Coulter). Gating strategies are depicted in Supporting information Figure S5. Distinct cell subsets were quantified in terms of frequency rather than absolute numbers, since the latter are influenced by sampling manipulation procedures that are unrelated to biological patterns. Pre‐ and post‐treatment samples (*Post 3* vs *Pre*, *Post 6* vs *Post 3*, and *Post 6* vs *Pre*) were compared by using paired *t‐* test.

### 4.5 TCGA transcriptomic analysis

RNA‐seq data from TCGA clear cell RCC (KIRC) cohort were downloaded using TCGA Assembler (version 2.0.3). Data normalization was performed within lanes, and between lanes using R package EDAS Equation (version 2.12.0) and quantile normalized using preprocessCore (version 1.36.0). A single primary tumor sample was included per patient using the TCGA Assembler “ExtractTissueSpecificSamples” function. Previously flagged samples that did not pass assay‐specific QCs were excluded.^84^, ^85^ Data were log2 transformed with an (+1) offset. ES were calculated by ssGSEA on the log2 transformed, normalized gene‐level data. Gene sets to define ES of tumor‐associated pathways (n = 51) were used as described previously.^43^ For immune‐reated signatures, we used the same gene sets used for the PBMC analysis. ICR category was defined as previously described.^43^

The correlation between tumor‐associated signatures was calculated using Pearson test and plotted using “corrplot” (version 0.84).

### 4.6 Survival analysis

Clinical data from the TCGA RCC cohort (KIRC) were obtained from the TCGA Pan‐Cancer Clinical Data Resource.^86^ Patients were divided in tertiles based on enrichment scores of MDSC gene signatures (MDSC_INT, G‐MDSC, and MDSC_Angel). OS was used to generate Kaplan–Meier curves using a modified version of the ggkm function.^87^ Survival data were censored after a follow‐up period of 10 years. HR between groups, corresponding *p‐*values, and confidence intervals were calculated using cox proportional hazard regression with R package survival (version 2.41‐3). For each variable, the proportional hazard assumption (PHA) was checked by computing the Pearson product‐moment correlation (rho) between the Schoenfeld residuals and the transformed (log) survival time was computed using the cox.zph R function.^43^ The cox proportional hazard models were stratified for variables with a significant violation of the PHA.

### 4.7 In vitro effect of pazopanib on immune cells

PBMCs from healthy donors, obtained from the INT Blood Bank upon informed consent, were sorted for CD14‐negative cells by CD14‐magnetic sorting beads (Miltenyi Biotech) and cultured for 3 days with CD3/CD28 activating beads (Dynabeads, Gibco) and 30 IU/mL IL‐2 (Proleukin, Clinigen Healthcare B.V.). For NK cells, PBMCs were further sorted for CD3‐negative cells and cultured 30 IU/mL for 5 days with IL‐2 (1000 IU/mL) and IL‐12 (10 ng/mL, R&D Systems). Pazopanib (Selleck, Houston, TX) was resuspended in 1% DMSO and added to the medium during the culture period at the indicated titration doses. Medium with the DMSO concentration corresponding to the highest (20 nM) drug concentration was used as control. MDSC‐like cells were generated from CD14^+^ monocytes isolated from peripheral blood of healthy donors and transfected with mimics of miRNAs associated with MDSC function, as recently reported by our team.^42^ Briefly, the cocktail of miR‐146a, miR‐155, miR‐125b, miR‐100, let‐7e, miR‐125a, miR‐146b, miR‐99b mimics (Qiagen), was admixed at 50 nM in HiPerfect transfection reagent (Qiagen) with monocytes for 4 h before the addition of FCS‐containing medium. After an overnight incubation, cells were washed, plated at 1 × 10^6^ cells/mL in fresh culture medium additioned or not with Pazopanib at 5 nM. Cells and supernatants were collected 24 h later and tested by flow cytometry for the phenotypic profile and IL‐6 and CCL2 cytokine secretion by Cytokine Bead Array (CBA) (BD Biosciences), respectively. For T, NK cell and MDSC phenotyping, the same mAb panel applied to flow cytometry for PBMC profile of patients treated with Pazopanib was utilized. Student's paired *t*‐test was applied to evaluate statistical significant differences in pazopanib‐treated versus untreated cells.

## AUTHOR CONTRIBUTIONS

D.R. contributed to the conception and design of the work, data acquisition and data interpretation, performed data analysis, and drafted the manuscript. E.V. contributed to the conception and design of the work, data acquisition and data interpretation, and drafted the manuscript. V.H. contributed to the data analysis, interpretation of data, and revision of manuscript. A.C. contributed to the analysis and revision of manuscript. P.S. contributed to the analysis and revision of manuscript. L.C. contributed to the analysis and revision of manuscript; F.B. contributed to the analysis and revision of manuscript; R.R. contributed to the analysis and revision of manuscript; M.D. contributed to the analysis and revision of manuscript; L.L. contributed to the analysis and revision of manuscript; V.V. contributed to the analysis and revision of manuscript; M.R. contributed to the analysis and revision of manuscript; J.R. contributed to the acquisition and analysis, interpretation of data, and revision of manuscript; C.C. contributed to the analysis and revision of manuscript; D.C. contributed to data interpretation, and substantively revised the manuscript; G.P. contributed to data interpretation, and substantively revised the manuscript; D.B. conceived and designed the study, contributed to the acquisition, data analysis and data interpretation, supervised the analysis and drafted the manuscript.

L.R. conceived and designed the study, contributed to the acquisition, data analysis and data interpretation, supervised the analysis and drafted the manuscript.

## AVAILABILITY OF DATA AND MATERIAL

Raw expression and normalized data matrix have been deposited at NCBI's Gene Expression Omnibus database (http://www.ncbi.nlm.nih.gov/geo/), with accession numbers GSE146163.

## COMPETING INTERESTS

EV reports personal fees for advisory boards from Pfizer, Ipsen, Novartis outside of the submitted work. No potential conflicts of interest were disclosed by the other authors.

## Supporting information

Figures

Table1

Table2

Table3
