# Supplementary material for: Integrated transcriptional‐phenotypic analysis captures systemic immunomodulation following antiangiogenic therapy in renal cell carcinoma patients
Source: Clin Transl Med. 2021 Jun 20;11(6):e434. doi: 10.1002/ctm2.434 (PMC8214860; doi:10.1002/ctm2.434)
Supplement: Supplementary file 1 — Figures [file CTM2-11-e434-s003.pdf]

A. **NK cell signaling pathways (3 vs 0)**

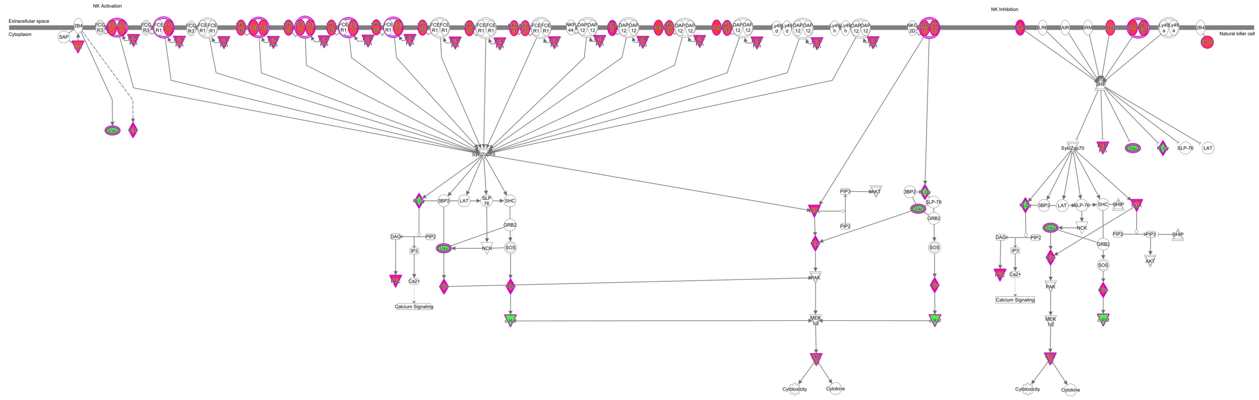

B. **Antigen presentation pathway (6 vs 3)**

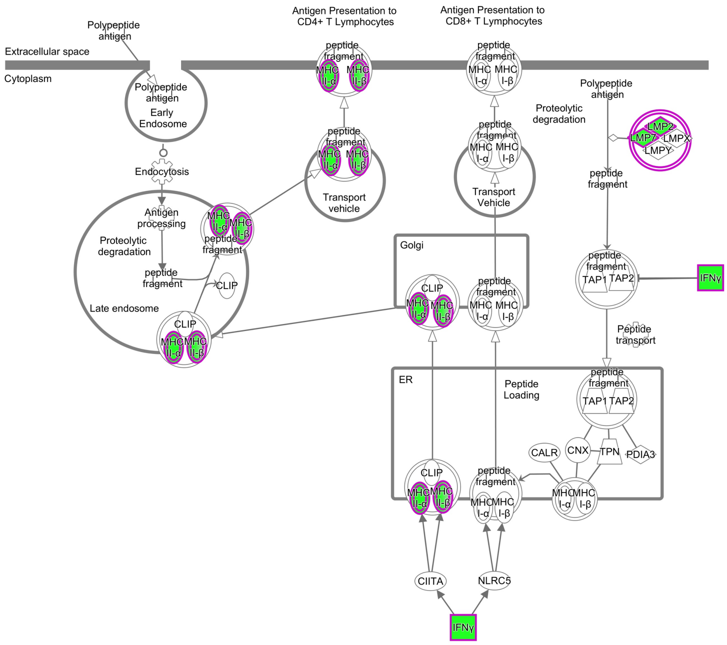

C. **Cross talk between dendritic cells & NK cells(6 vs 0)**

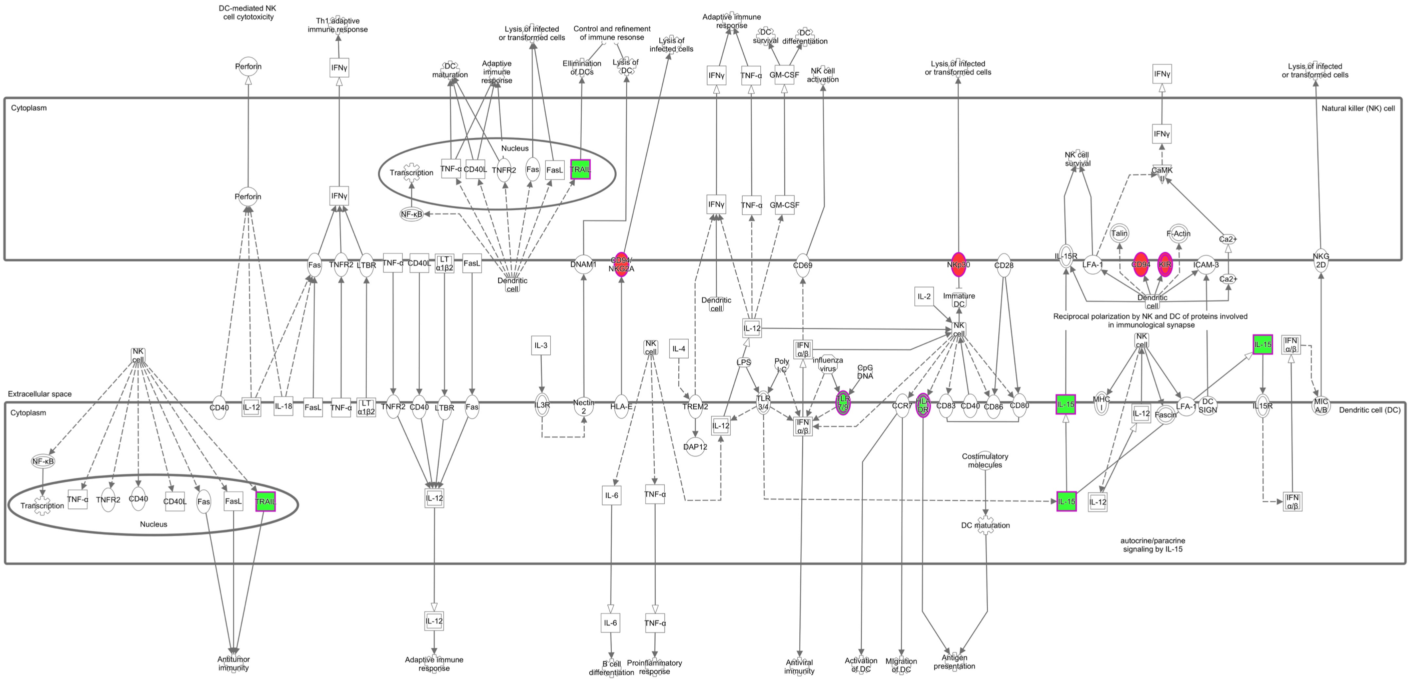

Supplementary Figure 1 : Top pathway in each time point comparison.

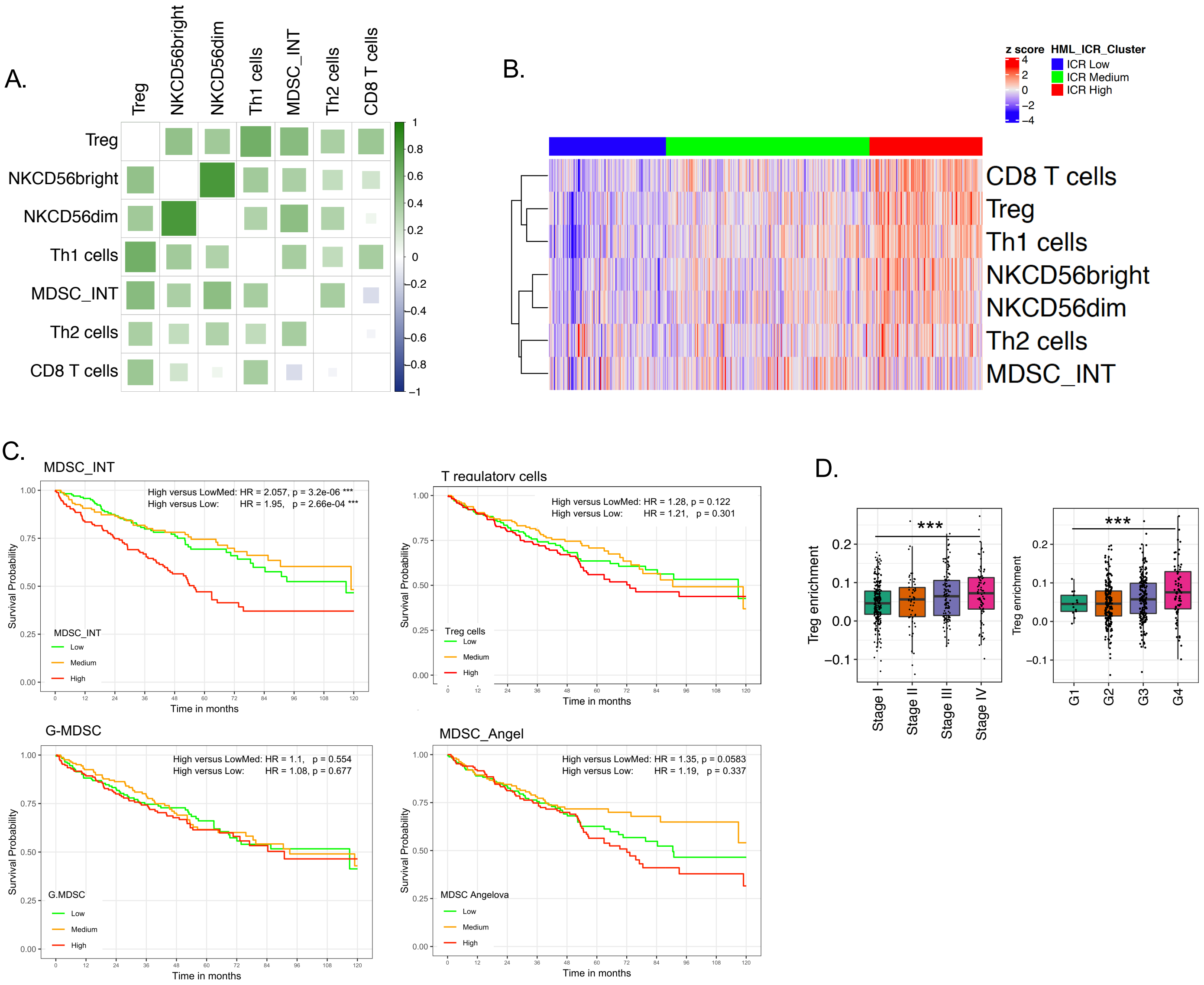

Supplementary Figure 2 : Prognostic value of MDSC, T cell and NK cell signatures. (A) Pearson correlation matrix of enrichment scores of MDSC_INT, NK and T cells signatures. (B) Heatmap of deconvolution immune cell type population. ICR category are defined as described elsewhere (Roelands et al, JITC 2020). (C) Kaplan Meier curves showing overall survival (OS) of patients within tertiles of MDSC_INT, granulocytic MDSC, MDSC Angelova, and reg scores. Cox proportional hazards statistics of the high versus LowMed (combination of intermediate and low tertiles) and High versus Low tertiles are shown. (D) Boxplots of Treg enrichment scores by AJCC pathologic (left) stage and (right) histological grade. t-test: *** represent *p* < *0.005, and * represent p < 0.05*.

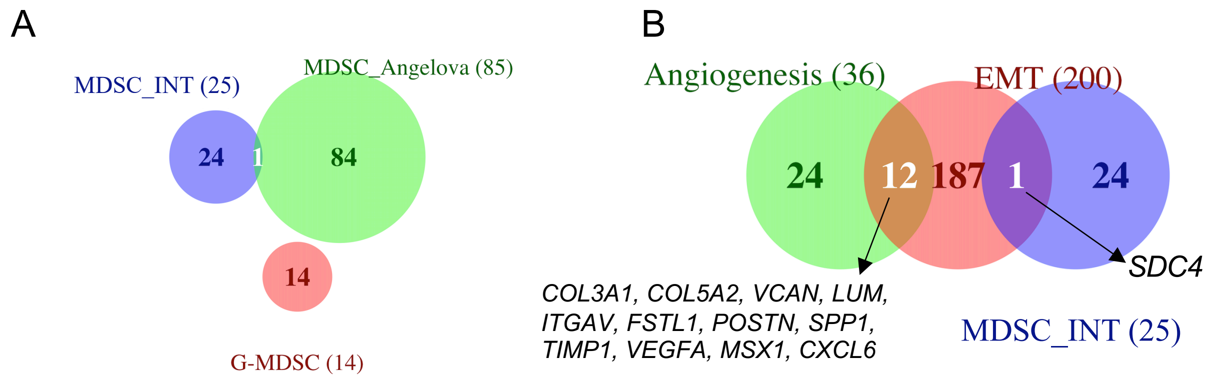

Supplementary Figure 3: Venn diagrams of intersection between MDSC signatures; MDSC_INT, MDSC_Angelova and G-MDSC (A) , and MDSC_INT and Cancer hallmark pathways (Angiogenesis and Epithelial mesenchymal transition) (B) . The total number of genes in each area is reported between the parentheses.

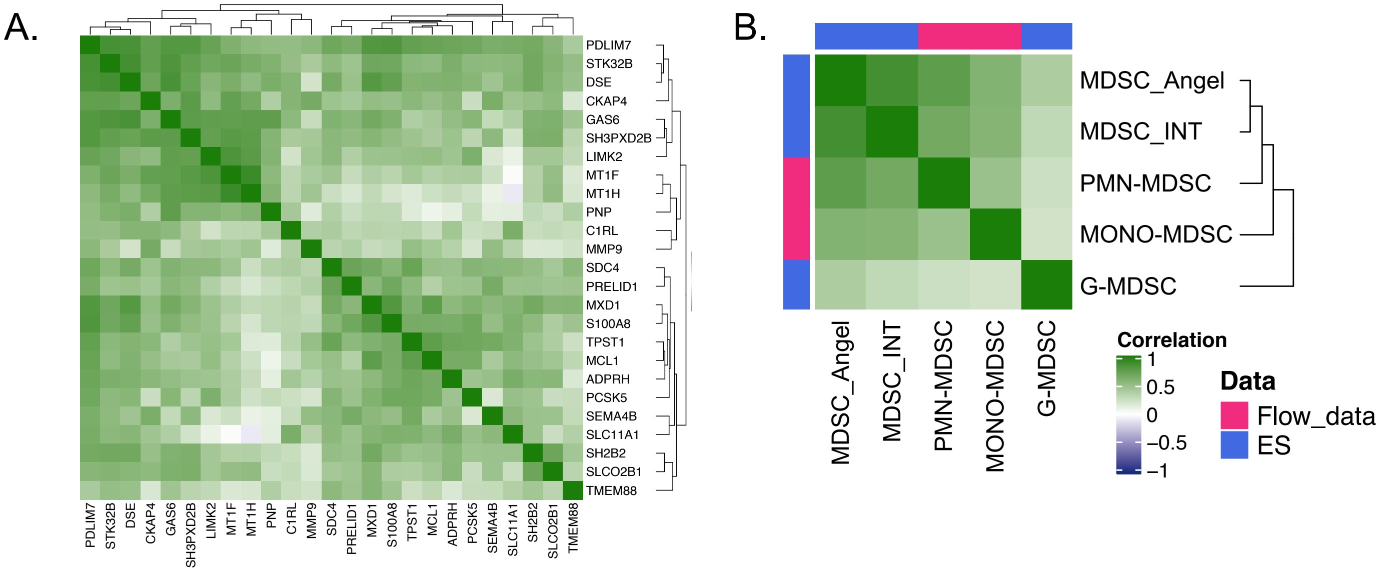

Supplementary Figure 4 : Pairwise Pearson correlation matrix between (A) 25 genes of the top r^2 (kmean1) of top 100 up-regulated genes from the data set obtained from human MDSC generated in vitro according to a model developed in our laboratory [Huber *et al*. JCI 2018]. **(B)** enrichment score of MDSC signature and percentage expression cell type by flow cytometry .

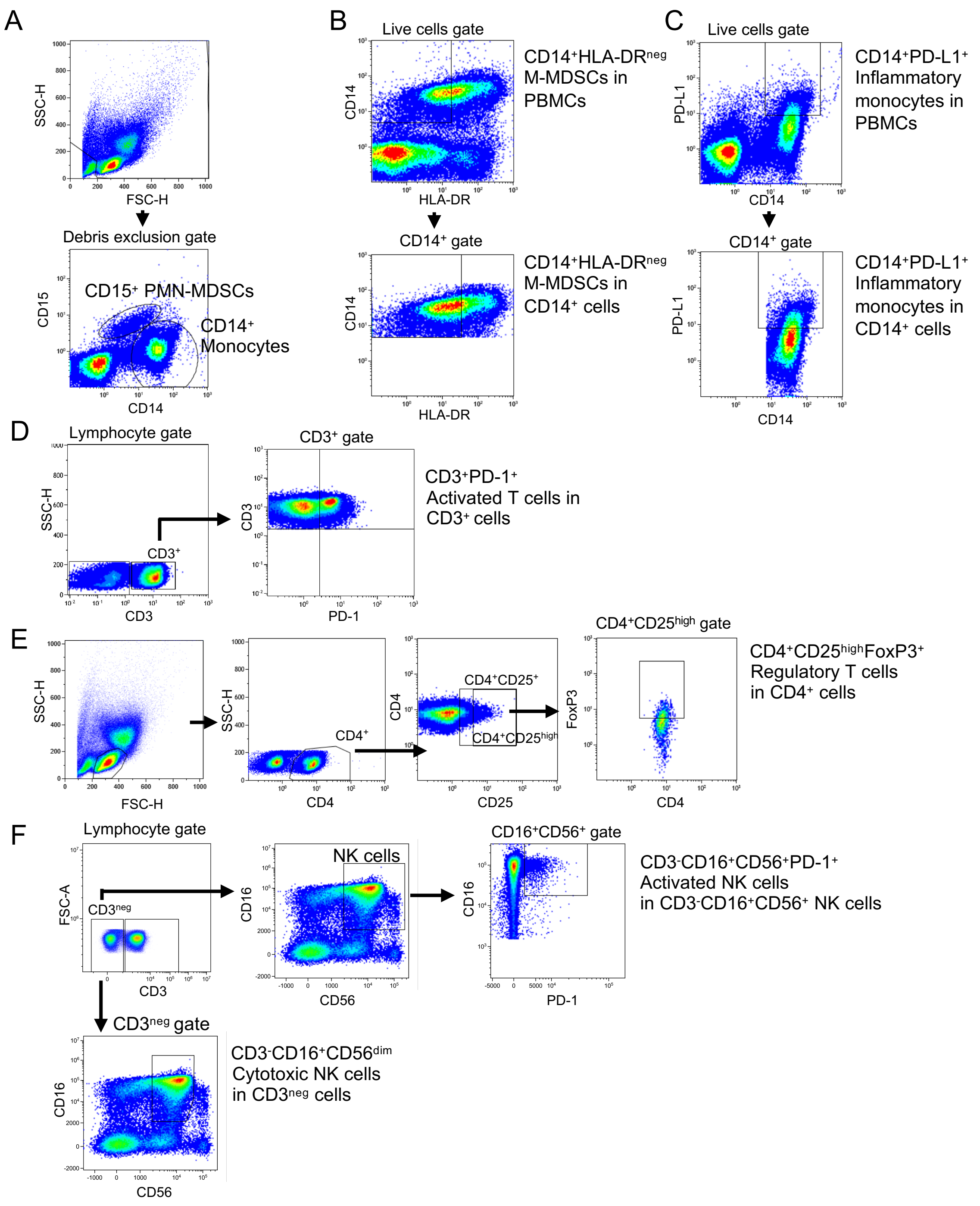

Supplementary Figure 5: Flow cytometry gating strategies. Myeloid cell populations: $CD15^{+}$ PMN-MDSCs, $CD14^{+}$ monocytes detected in the debris exclusion gate, (A); $CD14^{+}HLA\text{-}DR^{neg}$ M-MDSCs in PBMC (upper panel) and in $CD14^{+}$ cells (lower panel, B). The upper panel shows the setting of the HLA-DR gate in live cells (PBMC) to define the HLA-DR negative population based on FMO and internal reference; $CD14^{+}PD\text{-}L1^{+}$ inflammatory monocytes in PBMC (upper panel, gate set based on FMO) and in $CD14^{+}$ cells (lower panel, C). T and NK cell populations: $CD3^{+}PD\text{-}1^{+}$ activated T cells (D); $CD4^{+}CD25^{hi}$ $FoxP3^{+}$ regulatory T cells (E); $CD3^{-}CD16^{+}CD56^{+}PD\text{-}1^{+}$ activated NK cells and $CD3^{-}CD16^{+}CD56^{dim}$ cytotoxic NK cells (F).
